# A multimodal cortical network of sensory expectation violation revealed by fMRI

**DOI:** 10.1002/hbm.26482

**Published:** 2023-09-18

**Authors:** Miro Grundei, Timo Torsten Schmidt, Felix Blankenburg

**Affiliations:** ^1^ Neurocomputation and Neuroimaging Unit Freie Universität Berlin Berlin Germany; ^2^ Berlin School of Mind and Brain Humboldt Universität zu Berlin Berlin Germany

**Keywords:** connectivity, cross‐modal, mismatch negativity, mismatch responses, multisensory, P3, predictive processing, statistical regularity

## Abstract

The brain is subjected to multi‐modal sensory information in an environment governed by statistical dependencies. Mismatch responses (MMRs), classically recorded with EEG, have provided valuable insights into the brain's processing of regularities and the generation of corresponding sensory predictions. Only few studies allow for comparisons of MMRs across multiple modalities in a simultaneous sensory stream and their corresponding cross‐modal context sensitivity remains unknown. Here, we used a tri‐modal version of the roving stimulus paradigm in fMRI to elicit MMRs in the auditory, somatosensory and visual modality. Participants (*N* = 29) were simultaneously presented with sequences of low and high intensity stimuli in each of the three senses while actively observing the tri‐modal input stream and occasionally reporting the intensity of the previous stimulus in a prompted modality. The sequences were based on a probabilistic model, defining transition probabilities such that, for each modality, stimuli were more likely to repeat (*p* = .825) than change (*p* = .175) and stimulus intensities were equiprobable (*p* = .5). Moreover, each transition was conditional on the configuration of the other two modalities comprising global (cross‐modal) predictive properties of the sequences. We identified a shared mismatch network of modality general inferior frontal and temporo‐parietal areas as well as sensory areas, where the connectivity (psychophysiological interaction) between these regions was modulated during mismatch processing. Further, we found deviant responses within the network to be modulated by local stimulus repetition, which suggests highly comparable processing of expectation violation across modalities. Moreover, hierarchically higher regions of the mismatch network in the temporo‐parietal area around the intraparietal sulcus were identified to signal cross‐modal expectation violation. With the consistency of MMRs across audition, somatosensation and vision, our study provides insights into a shared cortical network of uni‐ and multi‐modal expectation violation in response to sequence regularities.

## Graphical abstract

Mismatch responses to tri‐modal roving stimulus sequences revealed a fronto‐parietal network of expectation violation shared across audition, somatosensation and vision. The intraparietal network hub showed specific sensitivity for cross‐modal predictability of stimulus transitions embedded in the multi‐modal sequence configuration.

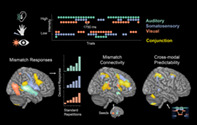

## 1 INTRODUCTION

The brain is constantly subjected to a multi‐modal stream of sensory inputs. As humans are encountering sensory information in an environment governed by statistical dependencies, the brain is engaging in probabilistic inference within and across sensory modalities (Barascud et al., 2016; Friston, 2005; Frost et al., 2015; Geisler, 2008; Gregory, 1980; Perruchet & Pacton, 2006; Summerfield & de Lange, 2014; Winkler et al., 2009). Neuronal mismatch responses (MMRs) to regularity violations such as the mismatch negativity (MMN; Näätänen et al., 1978, 2007) and the P3 (or P300; Polich, 2007; Squires et al., 1975; Sutton et al., 1965) have proven to provide valuable insights into the processing of probabilistic sensory input at various scales (Bekinschtein et al., 2009; Dehaene et al., 2015; Heilbron & Chait, 2018; Näätänen et al., 2001; Paavilainen, 2013; Schröger et al., 2014; Squires et al., 1975; Wacongne et al., 2011; Yaron et al., 2012). Although MMRs are among the most researched neural signatures (Näätänen et al., 2019; Polich, 2007), only few studies allow their direct comparison across sensory modalities and the mechanisms of modality specific and modality general MMRs to probabilistic *multi‐modal* inputs are largely unknown.

If sensory regularities, for example, repeating (standard) stimuli or stimulus patterns, are occasionally violated by rare (deviant) stimuli, brain signals typically recorded with EEG in form of MMRs can be observed. The most well‐known MMR is the auditory MMN, an early EEG component between ∼100 and 200 ms post‐stimulus onset which results from contrasting responses to deviant and standard stimuli. The MMN is elicited independent of attentional focus and task‐related top‐down processes (Alain & Woods, 1997; Näätänen et al., 1993; Ritter et al., 1999), even though attention can increase its amplitude (Trejo et al., 1995; Woldorff et al., 1991). Although primarily researched in the auditory modality, similar early MMN responses have been reported for other sensory modalities, including somatosensation (Andersen & Lundqvist, 2019; Gijsen et al., 2021; Hu et al., 2013; Kekoni et al., 1997; Ostwald et al., 2012; Shinozaki et al., 1998) and vision (Czigler, 2007; Kimura et al., 2011; Pazo‐Alvarez et al., 2003; Stefanics et al., 2014). The major neuronal generator of the auditory MMN is found in the auditory cortex where the exact location depends on the eliciting sound features and their complexity (Alho, 1995; Giard et al., 1990; Molholm et al., 2005; Sabri et al., 2004), with additional contributions from inferior frontal cortex (Deouell, 2007; Doeller et al., 2003; Molholm et al., 2005; Opitz et al., 2002; Rinne et al., 2005; Shalgi & Deouell, 2007). Similarly, the neuronal sources underlying the somatosensory MMN are found in primary and secondary somatosensory cortices (Akatsuka, Wasaka, Nakata, Kida, Hoshiyama, et al., 2007; Akatsuka, Wasaka, Nakata, Kida, & Kakigi, 2007; Andersen & Lundqvist, 2019; Butler et al., 2012; Gijsen et al., 2021; Grundei et al., 2023; Naeije et al., 2016; Ostwald et al., 2012; Spackman et al., 2010), in combination with inferior frontal cortex (Allen et al., 2016; Chen et al., 2008; Fardo et al., 2017; Grundei et al., 2023; Ostwald et al., 2012). The combination of sensory and frontal sources is also indicated to underlie the visual MMN (Hedge et al., 2015; Iglesias et al., 2013; Kimura et al., 2011; Pazo‐Alvarez et al., 2003; Yucel et al., 2007). Overall, ample evidence is pointing to modality‐specific MMN generators in sensory regions and more modality‐general contributions from (right inferior) prefrontal cortex.

A second well researched MMR is the P3, a later positive signal in response to stimulus deviance between 250 and 500 ms. The P3 is generally considered to be an attention‐dependent response (Duncan et al., 2009; Duncan‐Johnson & Donchin, 1982; Näätänen & Gaillard, 1983; Polich, 2007). While the earlier P3a sub‐component is task‐independent, drawing observers' attention to novel or unexpected stimuli (Escera et al., 2000; Friedman et al., 2001; Knight & Scabini, 1998), the later P3b response is more sensitive to task‐related target stimuli (Duncan et al., 2009; Polich, 2007). Although extensively researched in the auditory domain, the P3 is known for its modality independent characteristics (Linden, 2005; Polich, 2007) and has been equivalently reported for somatosensation (Andersen & Lundqvist, 2019; Gijsen et al., 2021; Ostwald et al., 2012; Shen et al., 2018a, 2018b; Yamaguchi & Knight, 1991, 1992) and vision (Conill, 1998; Duncan et al., 2009; Picton, 1992; Zhang et al., 2022), and has been described across senses in response to multi‐modal sequences (Grundei et al., 2023). The generating sources underlying the P3 are thought to be distributed in a fronto‐parietal network, involving inferior frontal, anterior cingulate and temporo‐parietal regions, with some indications for pronounced frontal contributions for the P3a and parietal dominance for the P3b (Linden, 2005; Polich, 2007). Thus, research on the P3 supports a modality‐general role for fronto‐parietal network activations in the processing of expectation violation and novelty alerting (Linden, 2005; Polich, 2007; Squires et al., 1975; Sutton et al., 1965).

Brain connectivity analyses based on fMRI and electrophysiological recordings support comparable network mechanisms underlying MMRs across modalities. Studies using dynamic causal models (DCM) indicate modulations in bidirectional connectivity in a fronto‐sensory network hierarchy underlying MMRs in the auditory (Chennu et al., 2016; Garrido et al., 2007; Garrido et al., 2008; Garrido, Kilner, Kiebel, & Friston, 2009; Hughes et al., 2013; Phillips et al., 2015; Phillips et al., 2016) and somatosensory modality (Allen et al., 2016; Fardo et al., 2017), and propose that feedforward and feedback connections carry sensory errors and top‐down expectations respectively. Similar mechanisms are hypothesized for visual MMRs (Stefanics et al., 2014). Moreover, parietal contributions to this network have been indicated for MMRs in somatosensation via DCM (Fardo et al., 2017) as well as to auditory MMRs via psychophysiological interaction (PPI) analyses in fMRI (Uhrig et al., 2014). Beyond the auditory modality only few studies have investigated MMR‐related connectivity modulations (Allen et al., 2016; Fardo et al., 2017; Kellermann et al., 2017; Sherman et al., 2016), and findings for multi‐modal inputs are largely lacking.

In a series of seminal articles, Downar and colleagues have investigated MMRs to multi‐modal stimulus sequences (Downar et al., 2000, 2001, 2002). These studies provided first indications for a multi‐modal mismatch network of sensory specific activation in hierarchically higher sensory cortices and shared activations in inferior frontal and temporo‐parietal regions in line with converging evidence from the auditory modality (Chennu et al., 2016; Dürschmid et al., 2016; El Karoui et al., 2015; Phillips et al., 2015; Phillips et al., 2016; Uhrig et al., 2014). A meta‐analysis of neuroimaging studies corroborated these reports, revealing similar fronto‐parietal MMR‐related activation for the auditory and visual modality (Kim, 2014). Furthermore, current research on statistical learning points to domain‐general computations underlying associative and probabilistic learning across different senses (Frost et al., 2015; Saffran & Thiessen, 2007). It has been shown that intraparietal and inferior frontal cortex encode the abstract structure of auditory and visual sequence patterns (Dehaene et al., 2015; Planton & Dehaene, 2021; Wang et al., 2015; Wang et al., 2019), highlighting their modality independent role during the extraction of regularities. Moreover, multi‐modal integration has been indicated during sequence processing (Bresciani et al., 2006, 2008), in terms of modulatory influences on the MMN (Besle et al., 2005; Butler et al., 2012; Friedel et al., 2020; Kiat, 2018; Zhao et al., 2015) and during multi‐modal causal inference in a fronto‐parietal network (Cao et al., 2019; Noppeney, 2021). Therefore, current research suggests a role for modality general fronto‐parietal activation during probabilistic sensory inference and it is of great interest to characterize the processing of multi‐modal statistical regularities and the underlying network in more detail.

An early finding, which has become a focus of the research on statistical sensory learning, is the modulation of the auditory MMN by standard repetition, that is, its amplitude increase with the number of prior standard presentations (Cowan et al., 1993; Haenschel et al., 2005; Imada et al., 1993; Sams et al., 1983). More recently, such modulation was shown beyond the auditory modality for both, MMN and P3 (Gijsen et al., 2021; Grundei et al., 2023). Although discussion persists about the contribution of stimulus specific adaptation in early sensory regions to these effects (Jääskelainen et al., 2004; May & Tiitinen, 2010), evidence converges to the view that increasing top‐down modulations in response to the repeating stimulus accounts best for this observation (Auksztulewicz et al., 2017; Auksztulewicz & Friston, 2016; Baldeweg, 2006; Ewbank et al., 2011; Garrido, Kilner, Stephan, & Friston, 2009; Langner et al., 2011; Summerfield et al., 2008; Todorovic & de Lange, 2012), particularly as studies have highlighted the dependence of MMRs on stimulus expectation based on statistical regularities as opposed to mere changes in stimulus properties (Bendixen et al., 2012; Heilbron & Chait, 2018; Wacongne et al., 2011). Computational modeling of EEG dynamics has indicated that probabilistic learning of environmental statistics underlies MMRs in the auditory (Lecaignard et al., 2022; Lieder, Daunizeau, et al., 2013; Weber et al., 2020), somatosensory (Gijsen et al., 2021; Grundei et al., 2023; Ostwald et al., 2012) and visual modality (Stefanics et al., 2018). This view is supported by studies showing deviant responses to abstract rule violations (Näätänen et al., 2010; Paavilainen, 2013; Schröger et al., 2007), unexpected stimulus repetitions (Alain et al., 1999; Horvath & Winkler, 2004; Macdonald & Campbell, 2011; Nordby et al., 1988) and unexpected stimulus omissions (Bendixen et al., 2009; Chennu et al., 2016; SanMiguel et al., 2013; Suda et al., 2022; Yabe et al., 1997). Such key properties of the auditory MMN (Wacongne et al., 2012) have similarly been reported for the somatosensory (Andersen & Lundqvist, 2019; Naeije et al., 2018) and the visual MMN (Czigler, 2007; Czigler et al., 2006) as well as for the (auditory) P3 (Duncan et al., 2009; Prete et al., 2022). Furthermore, it has been proposed that the deviance detection system of MMN and P3 differentially responds to expectation violation to sequence regularities on different levels of complexity. Studies employing the “local–global” paradigm (Bekinschtein et al., 2009) in which stimulus sequences are defined by local regularities (e.g., the tendency of a stimulus to repeat) and additional global regularities (e.g., every fifth stimulus in a repeated sequence is a deviant) show that the MMN is only elicited by the local regularity violations whereas the later P3 is additionally sensitive to violations of the global deviant regularity (Bekinschtein et al., 2009; Chennu et al., 2013; Chennu et al., 2016; Dürschmid et al., 2016; El Karoui et al., 2015; King et al., 2014; Niedernhuber et al., 2022; Shirazibeheshti et al., 2018; Wacongne et al., 2011). Strikingly, this dichotomy for MMRs was recently shown to hold for the auditory, somatosensory and visual modality alike (Niedernhuber et al., 2022). Evidence converges to the view that the MMN, induced by local deviants primarily activates sensory regions, while the P3 MMR after global deviance is accompanied by frontal (Chao et al., 2018; Chennu et al., 2013; El Karoui et al., 2015) and fronto‐parietal activations (Bekinschtein et al., 2009; Uhrig et al., 2014), in line with the neuronal sources thought to underlie the P3 (Linden, 2005; Polich, 2007). These results suggest that the P3 reflects expectation violation on a more global scale of sequence processing, indicating increasing levels of information integration in the hierarchy of a putative mismatch network.

Mechanistic accounts of universal principles of perception and perceptual learning in the brain, such as predictive processing (Clark, 2013; Friston, 2005, 2010), imply a modality independent role for MMRs reflecting error signals during expectation violation. Under such a view, the brain maintains a generative model of its environment which is continuously updated by comparing incoming sensory information with model predictions on different levels of hierarchical cortical organization (Friston, 2005; Kiebel et al., 2008; Mumford, 1992; Rao & Ballard, 1999). MMRs are interpreted as signatures of sensory prediction error in response to violations of top‐down predictions (Friston, 2005; Garrido, Kilner, Stephan, & Friston, 2009; Winkler & Czigler, 2012). Predictive coding models can account for key features of MMRs (Heilbron & Chait, 2018; Lieder, Stephan, et al., 2013; Wacongne et al., 2012) and the dichotomy of MMN and P3 identified by the local–global paradigm is thought to reflect differential processing stages in a predictive hierarchy operating on different levels of complexity and information integration (Bekinschtein et al., 2009; Chennu et al., 2016; Dürschmid et al., 2016; Uhrig et al., 2014; Wacongne et al., 2011). Similarly, the repetition modulation of MMRs reflect prediction error responses scaled by an increasing sensory expectation to repeat the current stimulus train (Auksztulewicz & Friston, 2016; Baldeweg, 2006). The results of prior empirical work, showing interactions in the fronto‐parietal and fronto‐sensory hierarchy of the cortex during mismatch processing across different modalities, are well in line with such mechanistic predictive processing accounts of MMR generation (Garrido, Kilner, Stephan, & Friston, 2009; Heilbron & Chait, 2018; Wacongne et al., 2012).

Overall, comparable dynamics of brain responses reflecting expectation violation within and across sensory modalities at different scales of complexity are of great interest for a mechanistic understanding of MMRs and fMRI investigations complementing the large body of work done in EEG remain rare. In the current study, we use a tri‐modal version of the roving stimulus paradigm (Grundei et al., 2023) to elicit MMRs in fMRI. The paradigm allows to study responses to rare stimulus transitions independent of their equiprobable features (Baldeweg et al., 2004; Cowan et al., 1993; Garrido et al., 2008). As such, we take into account the consensus that MMRs reflect mismatching sensory expectations rather than stimulus properties as well as a suggested fundamental role for probabilistic representations of stimulus *transitions* in sequence perception and statistical learning (Dehaene et al., 2015; Maheu et al., 2019; Meyniel et al., 2016; Mittag et al., 2016). Based on a probabilistic model, we generated multi‐modal sequences of low and high intensity stimuli for the auditory, somatosensory and visual modality which were governed by transition probabilities specifying cross‐modal conditional dependencies. As each stimulus transition was conditional on the prior tri‐modal stimulus configuration, the sequences exhibit global predictive properties in form of the multi‐modal context. The aim of the study was to identify a mismatch network of modality specific sensory cortices and modality independent hubs of mismatch processing in frontal and parietal cortices, as suggested by previous research (e.g., Downar et al., 2000). Moreover, we intended to show equivalences of MMRs across the senses, particularly in terms of the modulation of deviant responses with increasing expectation established by a local stimulus train (Baldeweg, 2006; Haenschel et al., 2005). Finally, our manipulation of the global stimulus predictability based on the other senses was expected to reveal potential higher‐level loci within the mismatch network sensitive cross‐modal expectation violation.

## 2 MATERIALS AND METHODS

Twenty‐nine participants with no history of psychiatric or neurological disorders completed the experiment (14 female; 15 male; age range 18–43, M = 28, SD = 5.9). Prior to the experiment, written informed consent was obtained from each participant. The study was approved by the ethics committee at the Freie Universität Berlin (003/2021).

**FIGURE 1:**
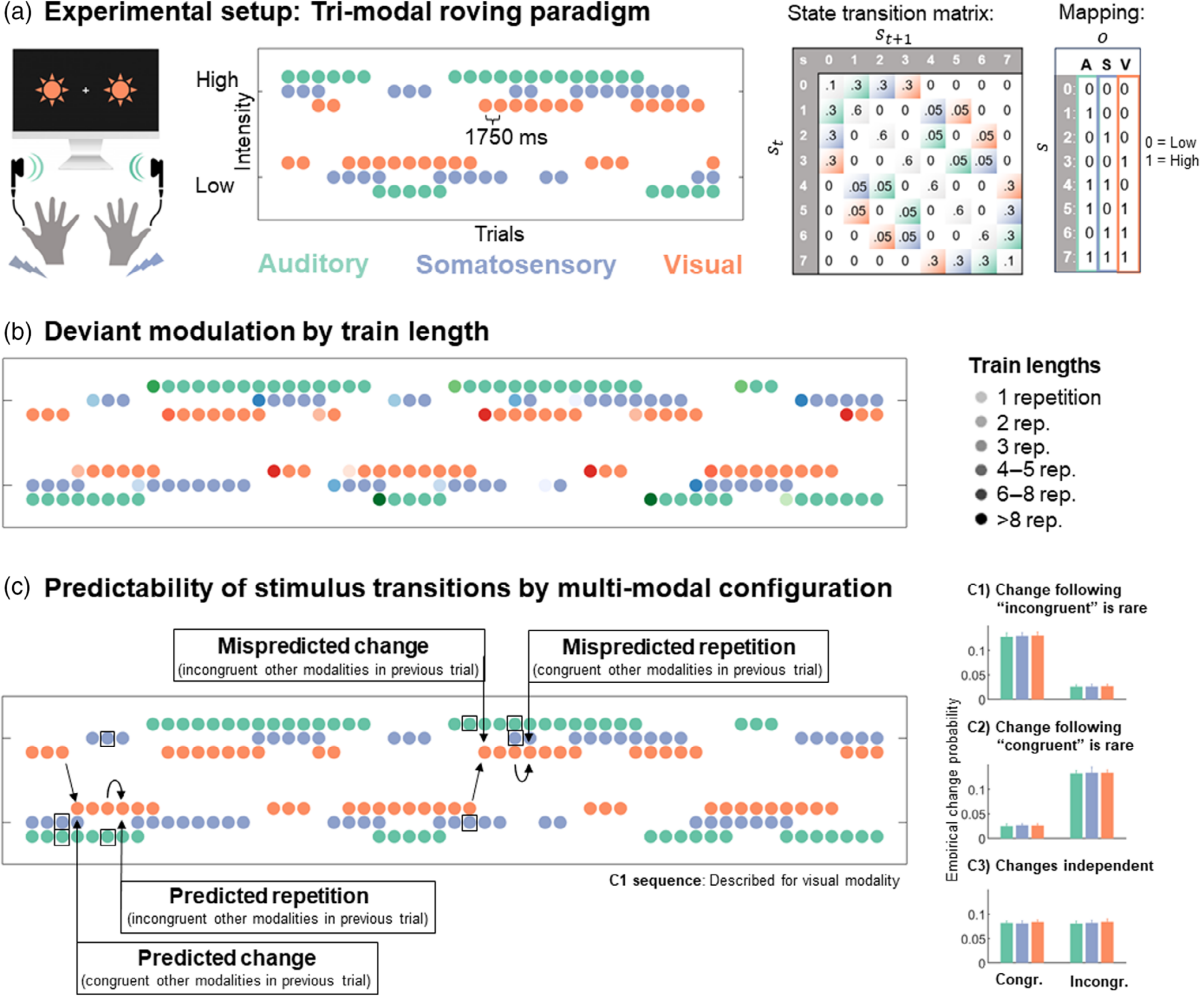
Experimental design. (a) Participants were presented with sequences of simultaneously presented bilateral auditory (A; green) “beep” stimuli, somatosensory (S; purple) electrical pulse stimuli and visual (V; orange) flash stimuli each at either low or high intensity. On consecutive trials, stimuli within each modality either repeated the previous stimulus intensity of that modality (standard) or alternated to the other intensity (deviant), corresponding to roving stimulus sequences for each modality (middle). Sequences were sampled according to a state transition matrix specifying the probabilities for states 0–7 at time *t* + 1 given the previous state $s_{t}$. Three different transition probability settings were used to define sequences for conditions C1, C2 and C3 (see Methods). Transition matrix here shown for probability setting C1. Light color shading depicts changes in a respective modality (A, S, V); light‐gray diagonal defines the probability of tri‐modal repetitions; white entries define the probability of multi‐modal changes which are set to zero. Each state maps to a specific tri‐modal observation (o) combination of low and high intensity stimuli (right table). (b) Deviant modulation by train length. Within the sequence of each modality stimulus repetitions form trains of standards of different lengths. The deviant following a specific standard train is labeled as falling in one of six categories depicted by color shading of the deviant (categories: 1, 2, 3, 4–5, 6–8, >8 repetitions). In our analyses we were interested in the modulation of the deviant response as a function of the standard train length preceding it. (c) Predictability of stimulus transitions by multi‐modal configuration. Left: Exemplary shown for a visual sequence with probability setting C1. Right: Empirical change probabilities for sequences of conditions C1, C2 and C3. Bars depict the mean percentage of occurrence ±standard error for the three modalities. In C1, if two modalities are congruent (both low or both high) a change in the third modality is more likely than when the other modalities are incongruent with each other (one low and one high, or v.v.). In C2, if two modalities are congruent a change in the third modality is less likely. In C3, changes are equally likely if the other two modalities are congruent or incongruent.

Participants underwent a multi‐modal version of the roving stimulus paradigm. Our paradigm, depicted in Figure 1a, consisted of simultaneously presented bilateral auditory (A), somatosensory (S) and visual (V) stimuli, which each alternated between two different intensity levels (‘low’ and ‘high’). The tri‐modal stimulus sequences originated from a single probabilistic model resulting in different combinations of low and high stimuli across the three modalities in each trial. The sequence generation process is described in detail for an EEG study in Grundei et al. (2023). In short, it consists of a state s at time t evolving according to a Markov chain ($p\left(s_{t}|s_{t-1}\right)$). Each state s represents 1 of 8 possible patterns as shown in Figure 1a with each state corresponding to a combination of three binary observations o. Therefore, each observation combination is conditional on the preceding observation combination ($p\left(o_{A,t},o_{S,t},o_{V,t}|o_{A,t-1},o_{S,t-1},o_{V,t-1}\right)$). For each modality, the binary observations have equal probability of occurrence $\left(p=.5\right)$ and stimulus transition probabilities were defined such that repetitions are more likely $\left(p=.825\right)$ than changes $\left(p=.175\right)$. For each modality, the overall configuration of change probabilities results in classic roving stimulus sequences, where trains of stimulus repetitions with different lengths alternate between the two stimulus intensities (depicted in Figure 1b). The range of train lengths was between 1 and 51 repetitions with an equal distribution across modalities (right skewed with expected value of 5 repetitions).

In each trial, for each of the three stimulus modalities, the other two modalities can be either both in low or high intensity (congruent) or one of them is low and the other high (incongruent). This property of the configuration of stimuli across the modalities was used to manipulate the predictability of stimulus transitions in the sequences. Three different types of stimulus sequences were generated with different settings (conditions C1, C2 and C3) determining the transition probabilities of each modality given the arrangement of the stimuli in the other two modalities (i.e., either congruent or incongruent; depicted in Figure 1c exemplified by a visual sequence for condition C1). Setting C1 defines higher change probability if the other two modalities are congruent (p(change|congruent) = 0.15, p(change|incongruent) = 0.025). Setting C2 defines lower change probability if the other two modalities are congruent (p(change|congruent) = 0.025, p(change|incongruent) = 0.15). Setting C3 defines equal change probability if the other two modalities are congruent or incongruent (p(change|congruent) = p(change|incongruent) = 0.0875). Per definition, the repetition probability follows the same principle such that for C1, p(repetition|congruent) = 0.85 and p(repetition|incongruent) = 0.975, for C2, p(repetition|congruent) = 0.975 and p(repetition|incongruent) = 0.85 and for C3, p(repetition|congruent) = p(repetition|incongruent) 0.925. Thus, in addition to the basic roving rule (i.e., repetitions are more likely than changes), for each modality, the settings C1 and C2 result in a tendency for stimuli to be more or less predicted in context of the two other modalities: In setting C1 a sequential stimulus tends to change more often (shows higher volatility) if accompanied by two congruent stimuli and tends to repeat more often (i.e., is more stable) if accompanied by two incongruent stimuli, and v.v. for C2. In other words, the multi‐modal context predicts the tendency for volatile and stable phases of the stimulus sequence. Using a terminology along the lines of Arnal and Giraud (2012), the resulting stimulus transitions for each modality within the different sequences can therefore be defined as being rather *predicted* (here: higher transition probability conditional on congruency/incongruency of the other modalities), rather *mispredicted* (here: lower transition probability) or *unpredictable* (here: equal transition probability) in context of the tri‐modal stimulus presentation. During each of 6 experimental runs, a sequence of stimuli with one of the three different probability settings (C1, C2, C3) was presented. For each participant unique sequences were sampled with randomly assigned conditions. The conditions were randomized across participants and a comparable number was presented overall, although condition C1 was presented more often (C1 = 63, C2 = 56, C3 = 55). In an alternative GLM setup presented in the Supplementary Materials which indicated highly comparable results (see Figure S1), the number of trials was balanced more rigorously, indicating that this slight imbalance in conditions is unlikely to have affected our main findings. The empirical change probabilities present in the applied sequences were ensured to be representative of the true underlying probabilities (see Figure 1c). Sequences were resampled if deviations were deemed too large. The range of deviations was set to ±0.005 for the probability differences in congruent/incongruent conditions. For the overall change probability as well as the probability of low and high intensity stimuli this range was set to ±0.025. Finally, a minimum number of one repetition was ensured by discarding trials during the sampling process which corresponded to changes following changes. Participants were uninformed about the sequence probabilities and they were task irrelevant. Upon completion of the experiment, participants were debriefed and asked if they noticed any regularities in the sequences.

### 2.1 Experimental setup

Each trial consisted of bilateral stimuli in three modalities (A, S, and V) that were presented simultaneously by triggering three instantaneous outputs of a data acquisition card (NI‐USB 6343; National Instruments Corporation, Austin, Texas, USA) with an inter‐stimulus interval (ISI) of 1750 ms. At each trial the device received and stored the corresponding stimulus waveform of each modality and released these to the stimulation devices upon a trigger signal, ensuring simultaneous stimulation.

Auditory stimuli were presented via in‐ear MRI compatible headphones (Siemens Healthcare GmbH, Erlangen, Germany) to both ears. The MRI internal auditory system was set to maximum and received auditory inputs from the data acquisition card consisting of sinusoidal waves of 500 Hz and 100 ms duration modulated in their amplitudes by two different voltage factors. These were individually adjusted with the participants prior to the experiment to obtain two clearly perceivable and distinguishable intensities (mean intensity across participants ± SD: low = 0.29 ± 0.3 V, high = 1.58 ± 1.28 V). As a different set of headphones was used for the first four participants, these were not included in the average intensity calculation.

Somatosensory stimuli were administered with two DS5 isolated bipolar constant current stimulators (Digitimer Limited, Welwyn Garden City, Hertfordshire, UK) via adhesive electrodes (GVB‐geliMED GmbH, Bad Segeberg, Germany) attached to the wrists of both arms to stimulate the median nerve. The stimuli consisted of electrical rectangular pulses of 0.2 ms duration, modulated by two different amplitudes. The two intensity levels were determined on an individual basis to obtain two clearly perceivable and distinguishable intensities (mean intensity across participants ± SD: low = 4.47 ± 0.99 mA, high = 7.47 ± 1.56 mA).

Visual stimuli were presented via light emitting diodes (LEDs) and transmitted through optical fiber cables (Loptec GmbH, Berlin, Germany). The LEDs were mounted 10 cm to the left and to the right of a fixation cross along the horizontal meridian (10°, eccentricity) presented on a display board at the base of the magnet bore at approximately 110 cm. The visual flashes consisted of rectangular waves of 100 ms duration which were modulated by two different voltage amplitudes (low intensity stimulus: 2.65 V, corresponding to approximately 0.4 lux; high intensity stimulus: 10 V, corresponding to approximately 91.5 lux). The visual stimuli were determined to be clearly perceivable and distinguishable by all participants so that no individual intensity adjustments were applied.

In each of 6 experimental runs, a sequence of 400 trials was presented. To ensure that participants maintained attention throughout the experiment and to encourage monitoring of all three stimulation modalities, participants were instructed to respond to occasional target questions (catch trials) via button presses with the foot. In six trials (<1%), randomly placed within each run, the fixation cross changed to one of the letters A, T or V followed by a question mark. This prompted participants to report if the most recent stimulus (before the appearance of the letter) in the auditory (letter A), somatosensory (letter T for “tactile”) or visual (letter V) modality was presented with low or high intensity. To minimize motion during responses, the hallux of the right foot was used by participants to press either a left or a right button on an MRI compatible button‐box, and the button assignment (left = low/right = high or left = high/right = low) was counterbalanced across participants.

### 2.2 fMRI data acquisition and preprocessing

Functional MRI data was acquired in 6 runs of 11.9 min on a 3 T Magnetom Prisma Fit Scanner (Siemens Healthcare GmbH, Erlangen, Germany) at the Center for Cognitive Neuroscience Berlin (CCNB), using a 64 channel head coil. Four hundred and seventy‐five functional volumes were acquired per run using a T2*‐weighted gradient‐echo EPI multiband 1 sequence (SMS factor = 3), with interleaved acquisition order and whole brain coverage (TR = 1.5 s; TE = 33 ms; 2.5 × 2.5 × 2.5 mm^3^ voxel; matrix size = 80 × 80, FOV = 200 mm, flip angle = 70°; 48 slices; gap = 10%). Additionally, a T1‐weighted MPRAGE with 208 sagittal slices, TR = 1930 ms, TE = 3.52 ms, 0.8 × 0.8 × 0.8 mm^3^ voxel size was acquired.

FMRI data were pre‐processed with SPM12 (Wellcome Trust Centre for Neuroimaging, Institute for Neurology, University College London, UK). Functional data were realigned to the mean image, normalized to MNI space using unified segmentation, interpolated to 2 × 2 × 2 mm^3^ voxel size, spatially smoothed with an 8 mm FWHM Gaussian kernel, and temporally detrended (Macey et al., 2004).

### 2.3 GLM analyses

Statistical analysis was performed according to a standard general linear model (GLM) approach with SPM12. For the experimental conditions, two separate first‐level GLMs were computed for each participant. Each analysis was applied on the full data set and all computed first level GLMs contained regressors of no interest for the motion parameters.

One first‐level GLM comprised a regressor modeling the onsets of all trials complemented with parametric regressors for each modality coding the trials with 1 and −1 for intensity (High > Low), mismatch responses (Deviants > Standards) and cross‐modal predictability (Mispredicted > Predicted). Correspondingly, three first‐level contrasts for each modality were computed. Please note that, contrary to conventional MMN analyses in EEG which only consider the pre‐deviant standard stimulus, in our analysis all standards were modeled in the GLM to avoid collinearity in the contrasts.

An additional subject‐level GLM was computed to test for parametric effects of deviant responses, dependent on the number of repetitions of standards before the deviant (train length; Figure 1b). We binned the deviant trials into six categories of train length (repetitions before the deviant: 1, 2, 3, 4–5, 6–8, >8) and created a model with a separate regressor for each of the train length categories for each modality. Computing separate contrast estimates for each corresponding train length allowed us to compute a linear contrast on the second level and plot the respective contrast estimates (shown in Results Figure 3).

The second level GLM analyses were performed as ANOVA models in terms of the flexible factorial design specification implemented in SPM. First‐level contrast images for each modality with one factor coding for modality, one factor coding for experimental condition were included supplemented with a subject factor. All second level conjunction analyses were computed as a conjunction against the global null hypothesis as implemented in SPM (Friston et al., 1999; Friston et al., 2005). Activation clusters were labeled according to anatomical and functional assignments provided by the cytoarchitectonic maps of the SPM Anatomy (Eickhoff et al., 2005).

### 2.4 Psychophysiological‐interaction analyses

To model changes in connectivity, we used PPI analyses as implemented in SPM (Friston et al., 1997). PPIs indicate if the contribution of one brain area to another changes significantly with an experimental factor and, as such, can be viewed as an event‐related connectivity measure. We tested for PPI‐connectivity changes during mismatch responses from seed regions in sensory cortices to the remaining brain voxels. The seed regions were based on the analysis of mismatch responses described above. From the seed regions we extracted time‐series data from volumes of interest defined as 8 mm radius spheres around peak voxels identified in the GLM analysis (somatosens: left *x* = −62, *y* = −16, *z* = 16; right: *x* = 58, *y* = −18, *z* = 22; auditory: left *x* = −60, *y* = −42, *z* = 12; right: *x* = 62, *y* = −38, *z* = 8; visual: left *x* = −44, *y* = −68, *z* = 2; right: *x* = 46, *y* = −58, *z* = 14). Following SPM's implementation of PPI analyses, for each modality, the interaction of the extracted source signal with the respective Deviants > Standards regressor of that modality was formed and included in a first level GLM analysis together with the source region's signal and all remaining experimental regressors. Subsequently, first level contrasts were computed for the interaction regressors and included in a second level GLM which included all PPI estimates (left and right of all modalities) and a subject factor.

## 3 RESULTS

### 3.1 Behavioral results

Participants performances in the “catch‐trials” indicates their ability to globally maintain their attention to the tri‐modal stimulus stream. Of the 70.9 ± 21% (M ± SD) responses that were given within the short response window (of 2.3 s), 73.5 ± 15.6% were correct with an average reaction time of 1.49 ± 0.21 s. One‐way repeated measures ANOVAs indicated no difference between modalities for response evaluation (*F*(2,50) = 0.02, *p* = .98) or reaction time (*F*(2,50) = 0.75, *p* = .48). Exclusion of a sample of participants (*n* = 5) showing bad response performance (who responded to ≤50% of questions in ≥50% of experimental runs) resulted in virtually identical fMRI results as presented below and no participants were removed from the analysis. Upon completion of the experiment, participants were debriefed and none of the participants identified the cross‐modal regularities in the sequences.

### 3.2 Mismatch responses across modalities

**FIGURE 2:**
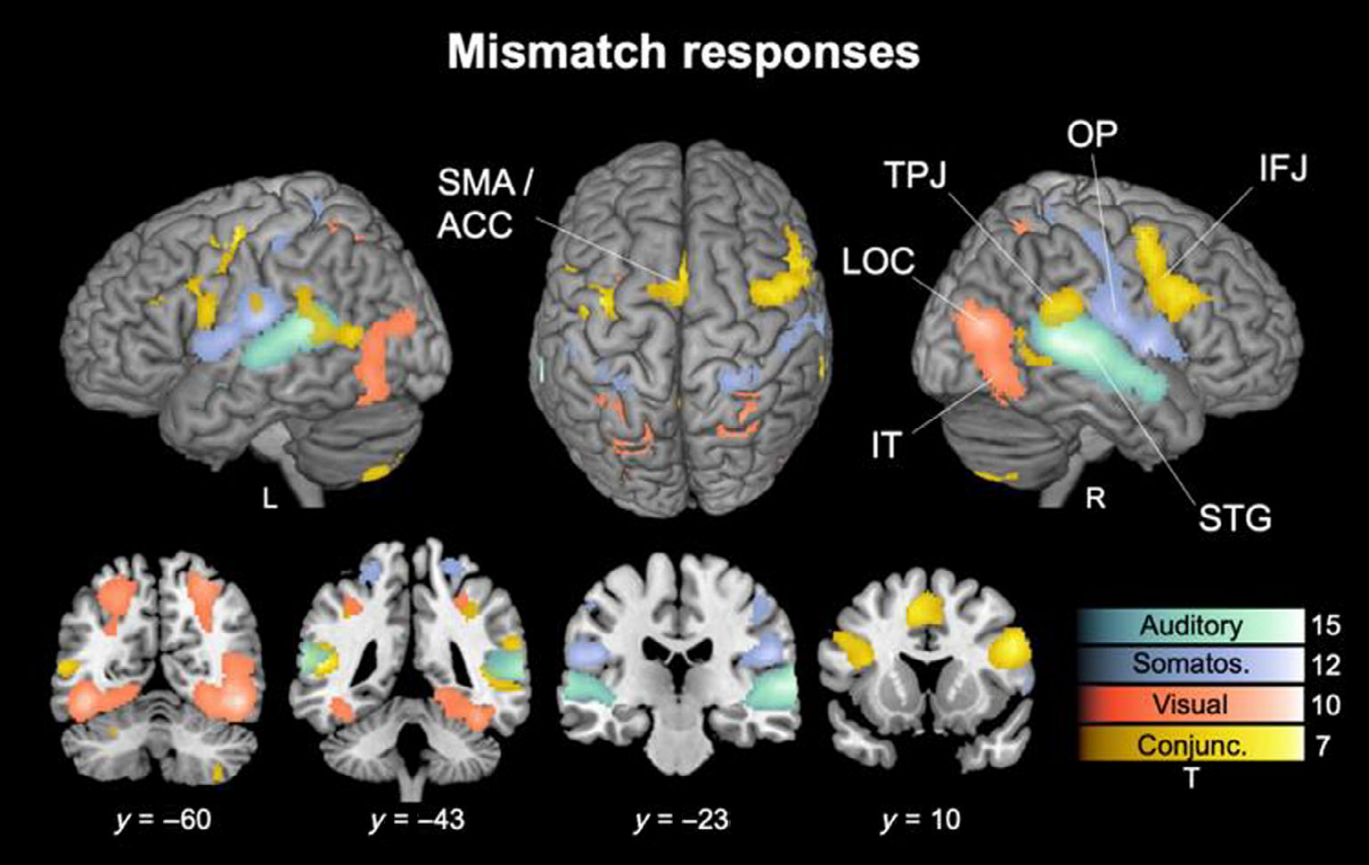
Mismatch responses. Contrasts of Deviants > Standards are shown for the auditory (green), somatosensory (purple) and visual (orange) modality as well as their conjunction (yellow), *p* < .05 FWE corrected on the peak level. Unthresholded SPMs are available at https://www.neurovault.org/collections/LECDZXPI. Abbreviations of region labels: IFJ: inferior frontal junction; IT: inferior temporal cortex; LOC: lateral occipital cortex; OP: Opercular cortex; SMA/ACC: (anterior) supplementary motor area/anterior cingulate gyrus; STG: superior temporal gyrus.

To reveal activation related to MMRs for each modality we computed the contrast Deviants > Standards against the same contrasts in the other two modalities, to delineate sensory specific activations. The results are presented in Figure 2, thresholded with *p* < .05 FWE corrected on the peak level and unthresholded SPMs of all results are available at https://www.neurovault.org/collections/LECDZXPI.

The auditory cluster spans bilaterally across the superior temporal gyrus (STG). Strongest activity was found in STG regions identified as auditory association areas in temporal cortex TE3 (right peak: *x* = 66, *y* = −32, *z* = 4, *t*‐value = 15.4; left peak: *x* = −64, *y* = −38, *z* = 12, *t*‐value = 13.5), temporal area TE4 in the upper bank of the superior temporal sulcus STS1 (right peak: *x* = 60, *y* = −20, *z* = 0, *t*‐value = 13.8; left peak: *x* = −62, *y* = −22, *z* = 2, *t*‐value = 10.1) and temporal area TE5 in the lower bank of the superior temporal sulcus STS2 (right peak: *x* = 54, *y* = 2, *z* = −12, *t*‐value = 10.7; left peak: *x* = −50, *y* = −12, *z* = −8, *t*‐value = 5.4). Moreover, some extensions into planum temporale and Heschl's gyrus were found on the left, encompassing primary auditory cortex in temporal area TE1 (left peak: *x* = −36, *y* = −32, *z* = 8, *t*‐value = 8.6).

The somatosensory cluster extends bilaterally across the postcentral gyrus and the operculum. Strongest activity was found in opercular cortex OP1, functionally identified as the secondary somatosensory cortex (SII; right peak: *x* = 48, *y* = −18, *z* = 18, *t*‐value = 10.2; left peak: *x* = −58, *y* = −18, *z* = 16, *t*‐value = 9.2), as well as the insular cortex (IC/Id4; right peak: *x* = 38, *y* = 0, *z* = 10, *t*‐value = 9.5; left peak: *x* = −40, *y* = −6, *z* = 10, *t*‐value = 7.9). The functional regions in postcentral gyrus were attributed to the primary somatosensory cortex (SI) in Brodmann areas BA2 (right peak: *x* = 26, *y* = −40, *z* = 68, *t*‐value = 7.4; left peak: *x* = −24, *y* = −42, *z* = 66, *t*‐value = 6.7) and BA3b (right peak: *x* = 48, *y* = −16, *z* = 36, *t*‐value = 6.2; left peak: *x* = −50, *y* = −16, *z* = 32, *t*‐value = 5.5).

The visual cluster extends bilaterally across the lateral occipital cortex (LOC) and inferior temporal cortex (IT) around the fusiform gyrus (FG). Strongest activity was found in FG (FG2; right peak: *x* = 46, *y* = −58, *z* = −14, *t*‐value = 11.9; left peak: *x* = −44, *y* = −64, *z* = −12, *t*‐value = 12.2) as well as LOC containing functional regions identified as higher order visual areas V4 (hOc4; right peak: *x* = 44, *y* = −74, *z* = 14, *t*‐value = 10.6) and V5 (hOc5; left peak: *x* = −44, *y* = −68, *z* = 2, *t*‐value = 7.5). Additional activation was found around the calcarine sulcus identified as visual areas V1 (hOc1; right peak: *x* = 6, *y* = −80, *z* = 6, *t*‐value = 5) and V2 (hOc2; right peak: *x* = 8, *y* = −84, *z* = 12, *t*‐value = 5.1).

The conjunction contrast across modalities shows bilateral clusters around the inferior frontal junction (IFJ) in the inferior frontal gyrus (IFG; right peak: *x* = 50, *y* = 12, *z* = 24, *t*‐value = 6.7; left peak: *x* = −50, *y* = −8, *z* = 26, *t*‐value = 5.3) and middle frontal gyrus (MFG; right peak: *x* = 42, *y* = 0, *z* = 52, *t*‐value = 5.8; left peak: *x* = −40, *y* = −4, *z* = 52, *t*‐value = 6.9), and is most pronounced on the right side where it extends into frontal operculum and IC (Id7; right peak: *x* = 32, *y* = 26, *z* = 2, *t*‐value = 2.7). Additional pronounced conjunction effects were found across the anterior portion of the supplementary motor area, extending into the anterior cingulate gyrus (SMA/ACC; left peak: *x* = −6, *y* = 10, *z* = 50, *t*‐value = 7.1), as well as bilaterally at the intersection of supramarginal gyrus (SMG), angular gyrus (AG) and superior and middle temporal gyrus (MTG) around the temporo‐parietal junction (TPJ; right peak: *x* = 54, *y* = −40, *z* = 22, *t*‐value = 5.2; left peak: *x* = −54, *y* = −44, *z* = 10, *t*‐value = 6.9).

### 3.3 Mismatch modulated connectivity

**FIGURE 3:**
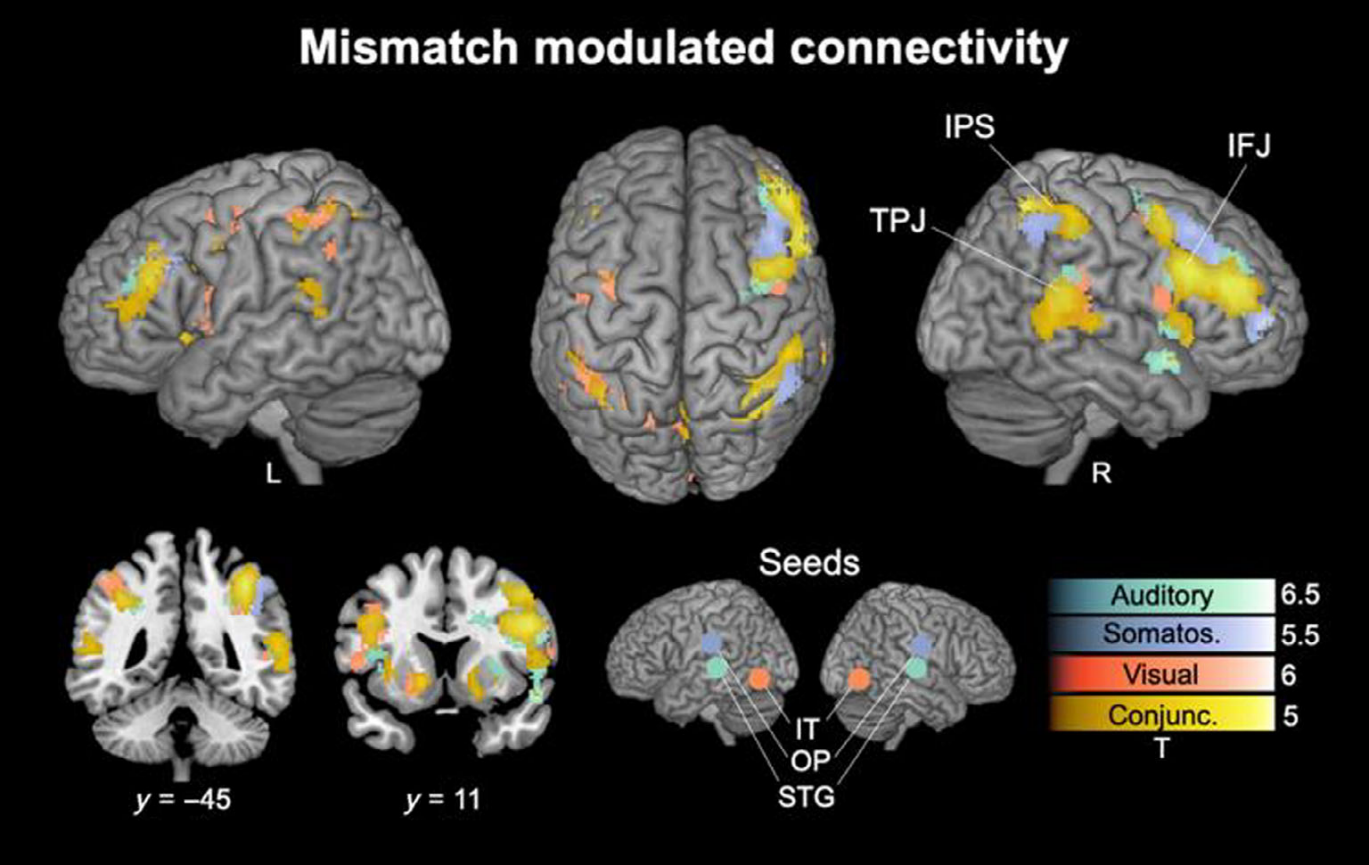
Mismatch modulated connectivity. Seed‐based psychophysiological interaction (PPI) connectivity analyses show connectivity modulations by the Deviants > Standards contrast from the three respective sensory seed regions to the rest of the brain. Clusters are shown for the auditory (green), somatosensory (purple) and visual (orange) modality and their conjunction (yellow). *p* < .05 FWE corrected on the cluster level. Unthresholded SPMs are available at https://www.neurovault.org/collections/LECDZXPI. Abbreviations of region labels: IFJ: inferior frontal junction; IPS: intraparietal sulcus; IT: inferior temporal cortex; OP: opercular cortex; STG: superior temporal cortex; TPJ: temporo‐parietal junction.

Results of seed‐based PPI‐connectivity analyses are presented in Figure 3, thresholded with *p* < .05 FWE corrected on the cluster level, showing connectivity increases from the three respective bilateral sensory seed regions to the rest of the brain, modulated by the mismatch contrast Deviants > Standards. Seed‐regions were in modality‐specific higher order sensory cortices of both hemispheres based on the strongest effects of the Deviants > Standards contrast presented above (see Figure 2), consisting of STG (auditory; left: *x* = −60, *y* = −42, *z* = 12; right: *x* = 62, *y* = −38, *z* = 8), OP/SII (somatosensory; left: *x* = −58, *y* = −18, *z* = 22; right: *x* = 58, *y* = −18, *z* = 22) and IT cortex (visual; left: *x* = −44, *y* = −68, *z* = 2; right: *x* = 46, *y* = −58, *z* = −14).

The conjunction contrast revealed a common increase in connectivity with brain areas found in the extended mismatch network comprised of bilateral frontal and temporo‐parietal regions with pronounced clusters on the right hemisphere. The frontal clusters are located bilaterally across the IFJ, including MFG (right peak: *x* = 44, *y* = 28, *z* = 22, *t*‐value = 3.6; left peak: *x* = −42, *y* = 4, *z* = 36, *t*‐value = 3.6) and IFG (right peak: *x* = 50, *y* = 12, *z* = 30, *t*‐value = 3.9; left peak: *x* = −40, *y* = 28, *z* = 20, *t*‐value = 3.4), with extensions into IC (Id6; right peak: *x* = 34, *y* = 20, *z* = 0, *t*‐value = 3.7; left peak: *x* = −34, *y* = 14, *z* = −2, *t*‐value = 3) and right frontal pole (FP) (peak: *x* = 42, *y* = 42, *z* = 22, *t*‐value = 4.1). The parietal clusters locate around the intraparietal sulcus (IPS; right peak: *x* = 36, *y* = −52, *z* = 50, *t*‐value = 4.6; left peak: *x* = −32, *y* = −56, *z* = 46, *t*‐value = 3.6), the TPJ (right peak: *x* = 54, *y* = 38, *z* = 18, *t*‐value = 3.2; left peak: *x* = −56, *y* = −42, *z* = 14, *t*‐value = 2.5) as well as precuneus (right peak: *x* = 8, *y* = −64, *z* = 44, *t*‐value = 3.4; left peak: *x* = −4, *y* = −62, *z* = 50, *t*‐value = 1.8).

### 3.4 Modulation of deviant response by train length

**FIGURE 4:**
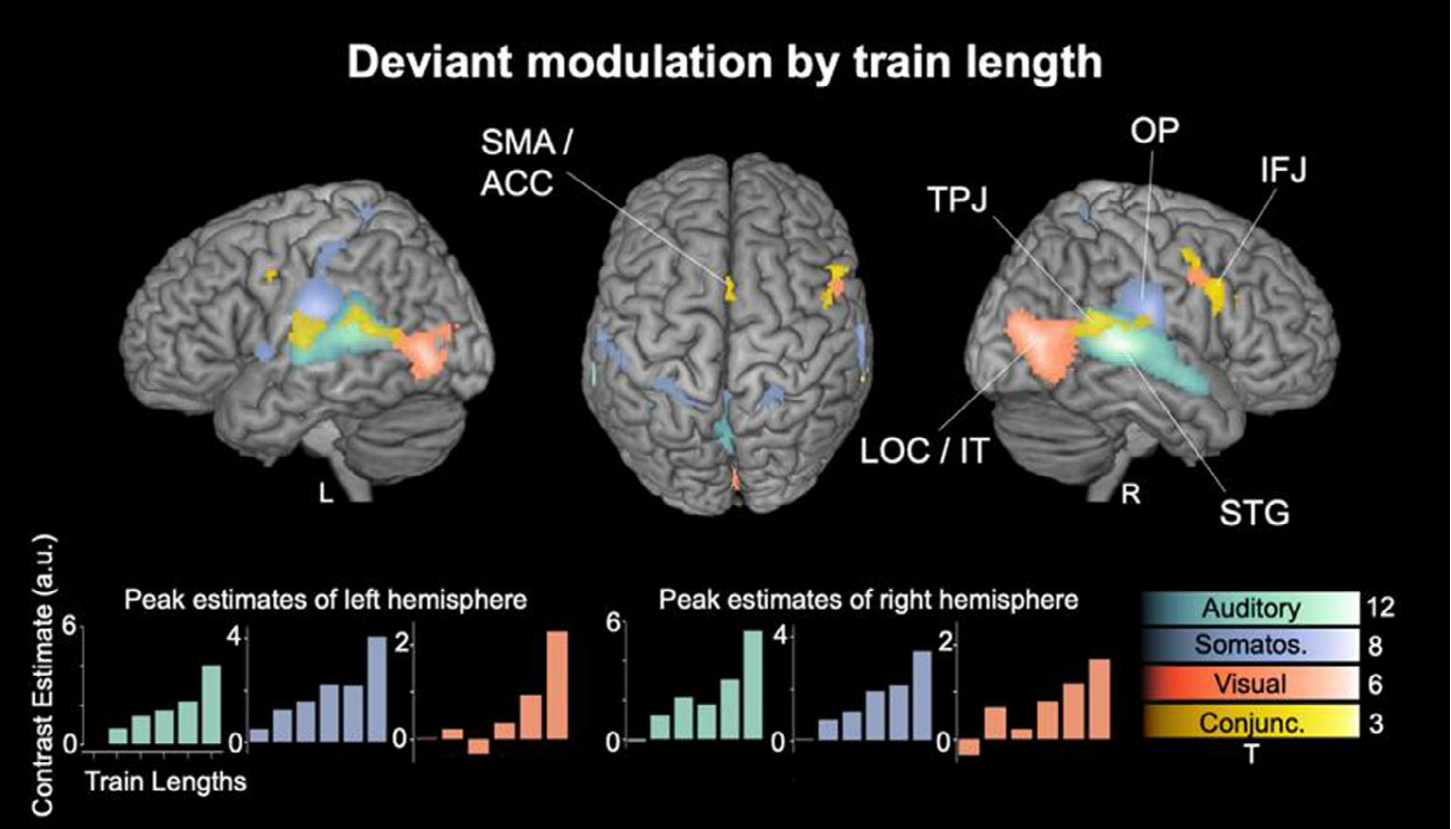
Modulation of deviant responses by prior repetition of standards. Parametric modulation of BOLD activity across six levels of stimulus train length (number of standards before the deviant; binned repetitions: 1, 2, 3, 4–5, 6–8, >8) for the auditory (green) somatosensory (purple) and visual (orange) modality and their conjunction (yellow). *p* < .05 FWE corrected on the cluster level. Unthresholded SPMs are available at https://www.neurovault.org/collections/LECDZXPI. Bottom row: The respective contrast estimates of the train length contrasts at the peak voxel of the main cluster of each modality (right and left). Abbreviations of region labels: IFJ: inferior frontal junction; IT: inferior temporal cortex; LOC: Lateral occipital cortex; OP: opercular cortex; SMA/ACC: (anterior) supplementary motor area/anterior cingulate cortex; STG: superior temporal cortex; TPJ: Temporo‐parietal junction.

The contrast of a parametric deviant increase with train length (defined by the number of standards presented prior to the deviant) for each modality is shown in Figure 4, thresholded with *p* < .05 FWE corrected on the cluster level. Significant clusters largely overlap with the effects in the mismatch network described above (Figure 2).

The auditory clusters are found bilaterally in STG with strongest activity in regions identified as auditory association areas TE3 (right peak: *x* = 66, *y* = −30, *z* = 6, *t*‐value = 12.5; left peak: *x* = −62, *y* = −36, *z* = 10, *t*‐value = 8.8) and TE4 (right peak: *x* = 52, *y* = −22, *z* = −2, *t*‐value = 7; left peak: *x* = −60, *y* = −16, *z* = 0, *t*‐value = 6.7) as well as the precuneus (right peak: *x* = 10, *y* = −74, *z* = −40, *t*‐value = 4.6; left peak: *x* = −6, *y* = −72, *z* = 46, *t*‐value = 4.9).

The somatosensory clusters primarily extend across the OP cortex including OP1, functionally corresponding to SII (right peak: *x* = 56, *y* = −16, *z* = 20, *t*‐value = 8.2; left peak: *x* = −58, *y* = −16, *z* = 20, *t*‐value = 7.1), and the IC (Id4; right peak: *x* = 40, *y* = −2, *z* = 10, *t*‐value = 7.6; left peak: *x* = −38, *y* = −6, *z* = 10, *t*‐value = 5.2). Additional activation was found in left postcentral sulcus identified as BA 2, containing SI (left peak: *x* = −48, *y* = −26, *z* = 42, *t*‐value = 4.3) and around the SMA/ACC (left peak: *x* = −2, *y* = 6, *z* = 36, *t*‐value = 4.3).

The visual clusters were found bilaterally in IT and FG (FG3; right peak: *x* = 32, *y* = −54, *z* = −14, *t*‐value = 5.2; left peak: *x* = −36, *y* = −56, *z* = −8, *t*‐value = 4.4), and most pronounced in LOC regions functionally corresponding to higher order visual areas V4 (hOc4; right peak: *x* = 48, *y* = −74, *z* = 8, *t*‐value = 5.8; left peak: *x* = −36, *y* = −80, *z* = 14, *t*‐value = 4.2) and V5 (hOc5; right peak: *x* = 42, *y* = −64, *z* = −2, *t*‐value = 5.8; left peak: *x* = −48, *y* = −70, *z* = 4, *t*‐value = 5.5). Additional clusters are observed in the calcarine sulcus identified as early visual areas V1/V2 (hOc1/hOc2; right peak: *x* = 4, *y* = −78, *z* = −12, *t*‐value = 4.6; left peak: *x* = −4, *y* = −86, *z* = 10, *t*‐value = 4.2) as well as round the right IFJ (right peak: *x* = 40, *y* = 8, *z* = 26, *t*‐value = 4.7).

The conjunction contrast across modalities shows most pronounced activation bilaterally around the IFJ, that is, IFG (right peak: *x* = 42, *y* = 4, *z* = 22, *t*‐value = 2.7; left peak: *x* = −46, *y* = 4, *z* = 30, *t*‐value = 2.3) and MFG (right peak: *x* = 48, *y* = 4, *z* = 42, *t*‐value = 2.1; left peak: *x* = −46, *y* = 2, *z* = 34, *t*‐value = 2.2), as well as the TPJ (right peak: *x* = 60, *y* = −42, *z* = 18, *t*‐value = 2.4; left peak: *x* = −54, *y* = −38, *z* = 18, *t*‐value = 2.9). Additional clusters are found around the SMA/ACC (right peak: *x* = 4, *y* = 4, *z* = 56, *t*‐value = 2).

Since the train lengths of a given modality are longer if accompanied by congruent other modalities in condition C1, and v.v. in C2, we performed an additional train length analysis using only trials of the unpredictable condition C3 (corresponding to around 1/3 of trials) with identical distributions of train lengths across congruent and incongruent trials. Although less significant (*p* < .001 uncorrected; likely due to the reduced number of trials), the results for trials of C3 (not shown) revealed the same main clusters sensitive to parametric increase in deviant responses as presented in Figure 4.

### 3.5 Cross‐modal predictability

**FIGURE 5:**
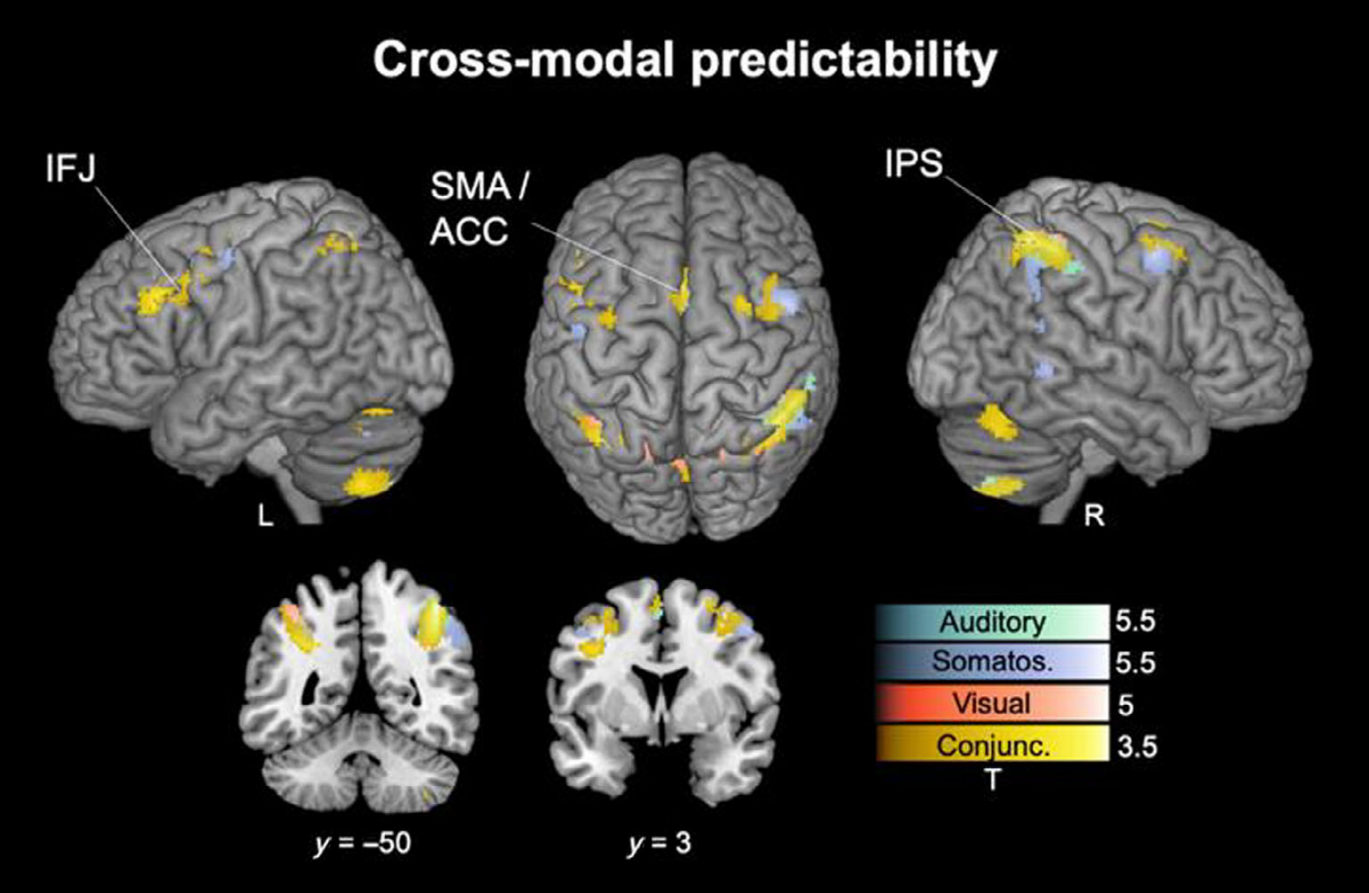
Cross‐modal expectation violation. Significant clusters of activation for the predictability regressor (contrasting Mispredicted > Predicted trials) for the auditory (green), somatosensory (purple) and visual (orange) modality and their conjunction (yellow). *p* < .05 FWE corrected on the cluster level. Unthresholded SPMs are available at https://www.neurovault.org/collections/LECDZXPI. Abbreviations of region labels: IFJ: inferior frontal junction; IPS: intraparietal sulcus; SMA/ACC: (anterior) supplementary motor area/anterior cingulate cortex.

Activations in response to mispredicted stimuli (with respect to the multi‐modal stimulus configuration) were identified by contrasting Mispredicted > Predicted trials for each modality. The contrast revealed increased activity in frontal and parietal clusters which are shown in Figure 5, thresholded with *p* < .05 FWE corrected on the cluster level. The clusters show overlap with clusters of the extended mismatch network described above. The auditory, somatosensory and visual modality showed the most pronounced clusters around the IPS, identified as a dorsal extension of the TPJ: The conjunction contrast across modalities shows bilateral clusters at the intersection of AG (right peak: *x* = 42, *y* = −50, *z* = 54, *t*‐value = 3.4; left peak: *x* = −30, *y* = −54, *z* = 34, *t*‐value = 2.6) and SMG (right peak: *x* = 50, *y* = −42, *z* = 52, *t*‐value = 2.9; left peak: *x* = −52, *y* = −44, *z* = 38, *t*‐value = 2.3) with extension into precuneus on the right (peak: *x* = 16, *y* = −64, *z* = 48, *t*‐value = 2.5). Additional clusters were found in the SMA/ACC (left peak: *x* = −2, *y* = 12, *z* = 50, *t*‐value = 2.8) and bilaterally around the IFJ, primarily in MFG (right peak: *x* = 40, *y* = 0, *z* = 54, *t*‐value = 1.9; left peak: *x* = −48, *y* = 26, *z* = 30, *t*‐value = 2.9).

## 4 DISCUSSION

Using a tri‐modal version of the roving stimulus paradigm in combination with fMRI, we induced MMRs in the auditory, somatosensory and visual modality corresponding to modality specific activation in sensory cortices and modality independent clusters of activation in inferior frontal and temporo‐parietal cortex. In addition to confirming initial fMRI work on multi‐modal MMRs (Downar et al., 2000), our results expand the previous description by showing deviance related modulation of PPI‐connectivity from each sensory region to the modality independent hubs of the putative cortical mismatch network. Moreover, across modalities, we showed increasing deviant responses within the identified network with prior standard repetition, most pronounced in higher order sensory regions. Strikingly, our novel experimental manipulation of cross‐modal stimulus predictability revealed a parietal contribution to mismatch processing selectively sensitive to cross‐modal regularity violation.

### 4.1 Modality specific activations in sensory cortices

In accordance with prior research, mismatch effects specific to the auditory input sequence were found in the STG (Doeller et al., 2003; Downar et al., 2000; Garrido, Kilner, Stephan, & Friston, 2009; Molholm et al., 2005; Näätänen et al., 2005; Opitz et al., 2002; Rinne et al., 2005; Yucel et al., 2005). Interestingly, the most pronounced activation was observed bilaterally in higher order auditory processing areas with only little overlap with primary auditory cortex, which is often specified in EEG source modeling as a separate source in Heschl's gyrus (e.g., Garrido, Kilner, Stephan, & Friston, 2009). In contrast, here we find sensory specific activations in secondary auditory areas across the STG (TE3) and in the upper (STS1/TE4) and lower bank of the superior temporal sulcus (STS2/TE5) which are considered high level auditory processing regions (Zachlod et al., 2020). Our results are in accordance with a recent comparative fMRI study showing STG (TE3) activation for MMR to intensity changes among other stimulus features (Zvyagintsev et al., 2020). Of the few studies, which have used the roving stimulus paradigm in fMRI, one showed activations of higher order auditory regions in STG for pattern (“what”) and location (“where”) changes (Altmann et al., 2007). Similarly, another study differentially located responses to duration and frequency deviants in STG as well as within inferior frontal and posterior parietal regions (Molholm et al., 2005). The authors find a tendency for left lateralization of duration deviants (temporal information) and right lateralization for frequency deviants (tonal information). Here, we supplement these findings with intensity changes in roving stimulus sequences resulting in sensory specific activations in higher order auditory and parietal regions with right hemispheric dominance. While it is obvious that primary auditory cortex contributes to the MMR as it receives sensory input signals, our results suggest that the primary generator signaling sensory specific mismatch lies in non‐primary auditory regions in STG. These results are in line with DCM studies modeling the STG as the intermediate stage of MMR processing, receiving feedforward input from primary auditory and feedback input from pre‐frontal cortex (Auksztulewicz & Friston, 2015; Chennu et al., 2016; Garrido et al., 2007; Garrido et al., 2008; Garrido, Kilner, Kiebel, & Friston, 2009; Phillips et al., 2015; Phillips et al., 2016) and support their interpretations of auditory MMRs reflecting prediction error signals in response to violation of top‐down predictions.

Mismatch effects specific to the somatosensory sequence were observed in SI and SII extending into IC. Most pronounced activation was found in SII and activation in SI was primarily found in BA1 and BA2 which are not major input regions (Purves et al., 2008). Previous research on somatosensory MMRs was primarily done with EEG and MEG, where source modeling suggests underlying neuronal generators in SI and SII (Akatsuka, Wasaka, Nakata, Kida, Hoshiyama, et al., 2007; Akatsuka, Wasaka, Nakata, Kida, & Kakigi, 2007; Andersen & Lundqvist, 2019; Butler et al., 2012; Gijsen et al., 2021; Grundei et al., 2023; Naeije et al., 2016, 2018; Ostwald et al., 2012; Spackman et al., 2010) and only very few studies investigated somatosensory MMRs with fMRI (Allen et al., 2016; Chen et al., 2008; Downar et al., 2000). Although the findings of Downar et al. (2000) in their tri‐modal fMRI study overlap largely with our mismatch network, their somatosensory specific activation was restricted to SII. Our results, on the other hand, showed additional activation in IC which was distinct from its multi‐modal activation in anterior portions (Id7) close to the IFJ cluster. Similarly, Chen et al. (2008) identified a comparable network of SII, IC and fronto‐parietal activations for somatosensory MMRs in fMRI to attended and unattended uni‐modal deviants during electrical median nerve stimulation (as used here), showing that SII activation was unmodulated by the attentional focus, in contrast to the higher‐level processing stages, indicating SII as a primary driver for the first stage of mismatch processing reflected in the MMN response. Moreover, the (right) IC has been suggested to be involved in the integration of ascending sensory information with descending signals from higher level regions in prefrontal cortex during somatosensory processing (Cerliani et al., 2012; Lovero et al., 2009; Seth et al., 2011), in line with our finding. Correspondingly, in a DCM study using somatosensory roving stimulus sequences, Allen et al. (2016) identified modulations in a network comprised of S1, IC and MFG during somatosensory mismatch processing, providing evidence for a role of IC in the coordination of hierarchical predictive interactions. In correspondence with the results of our PPI‐connectivity analysis, the authors showed increasing feedforward connectivity from somatosensory cortex to insular and prefrontal cortices for unexpected stimuli, while feedback projections were found between IC and somatosensory cortex, suggesting that the IC mediates the reciprocal exchange with hierarchically higher areas (Allen et al., 2016). Similarly, in another DCM study, Fardo et al. (2017) demonstrated that expectation violation reflected in somatosensory MMRs was accompanied by intrinsic modulations within the somatosensory system in SI and SII as well as extrinsic recurrent connectivity modulations between somatosensory, pre‐frontal and parietal regions. Taken together, our results are in support of interactions between SII and IC to underlie the sensory specific aspects of somatosensory mismatch processing.

Mismatch effects specific to the visual sequence were found in LOC and IT in hierarchically higher brain areas of visual processing such as V4, V5 and the fusiform gyrus. In parallel to the somatosensory modality, most previous research on visual MMRs was done using M/EEG recordings, generally indicating activation of the visual cortices (Czigler, 2007; Kimura et al., 2011; Pazo‐Alvarez et al., 2003). Nevertheless, some more direct evidence has been provided for hierarchically higher visual regions in occipito‐temporal cortex (Egner et al., 2010; Kimura et al., 2010; Stefanics et al., 2019; Urakawa et al., 2010; Yucel et al., 2007). A recent fMRI study identified MMRs to roving face stimuli (with emotion and color changes) in lateral occipital and posterior parietal cortex (Stefanics et al., 2019). Interestingly, although we used vastly different stimulus types (flashes as opposed to faces), we characterized highly similar higher order visual regions coding for the stimulus transitions. The authors identified a perceptual model reflecting precision weighted prediction errors as best explaining their results (Stefanics et al., 2018; Stefanics et al., 2019). Top‐down projections from higher cortical processing stages are thought to modulate responses in non‐primary visual areas in particular (Buffalo et al., 2010; Johnson et al., 2007; Kastner et al., 1998; Mohr et al., 2009) and visual research highlights the modulatory effects of sensory expectations in hierarchical visual processing (de Lange et al., 2018; Ferrari et al., 2022; Summerfield & de Lange, 2014; Summerfield & Egner, 2009). Given that activation in monkey IT cortex has shown to be reflective of expectation violation based on probabilistic information (Bell et al., 2016) with respect to the learning of transition probabilities (Meyer et al., 2014; Meyer & Olson, 2011), our results are well in line with current visual research on probabilistic sequence processing and in support of the notion that sensory specific visual MMRs reflect prediction error in higher visual areas violating top‐down predictions (Stefanics et al., 2014; Stefanics et al., 2018).

### 4.2 A modality general fronto‐parietal mismatch network

In addition to the sensory specific clusters, we identified a shared network of mismatch processing across modalities with activations around the IFJ, TPJ and around the SMA/ACC. Most pronounced activation was found in the right hemisphere, especially around the IFJ. The involvement of frontal cortex in deviance detection integrates well with evidence from MMN research where source modeling suggests a combined activation of sensory and (right) inferior frontal regions. Primarily researched in the auditory modality, this combination of neuronal generators has been repeatedly demonstrated using a large variety of electrophysiological methods such as EEG (Garrido et al., 2008; Garrido, Kilner, Stephan, & Friston, 2009; Giard et al., 1990; Giard et al., 1995; Rinne et al., 2000; Shalgi & Deouell, 2007), MEG (Rinne et al., 2000), intracranial EEG (Dürschmid et al., 2016; Phillips et al., 2016) as well as optical imaging (Tse et al., 2006; Tse et al., 2013). Comparable evidence is increasingly provided across modalities (Grundei et al., 2023), for example, in somatosensation (Allen et al., 2016; Fardo et al., 2017; Huang et al., 2005) and vision (Hedge et al., 2015; Tse et al., 2021).

Using fMRI, to our knowledge, only Downar et al. (2000) have investigated MMRs to similar multi‐modal sequences used here. The authors applied naturalistic stimuli, such as sounds of frogs and running water, visual shapes and tactile unilaterally applied brush strokes. Therefore, it is noteworthy that we replicated their neuroimaging results, showing the same uni‐ and multi‐modal activation patterns using a larger sample (*N* = 29 compared to *N* = 10) in response to stimulus sequences more commonly used in current MMR research. With a heavy focus on the auditory modality, a considerable number of studies have identified a similar fronto‐parietal mismatch network in uni‐modal fMRI experiments (Diekhof et al., 2009; Doeller et al., 2003; Molholm et al., 2005; Opitz et al., 2002; Rinne et al., 2005; Shalgi & Deouell, 2007). Particularly the IFJ and, as opposed to most implications from the EEG literature, additionally the TPJ have appeared as consistent findings in fMRI oddball‐studies across different modalities (Doricchi et al., 2022; Downar et al., 2000, 2001, 2002; Huang et al., 2005; Kim, 2014).

Given the temporal resolution of fMRI, it is impossible to isolate the MMN response from the effects of the later P3 MMR, although it is likely that fronto‐sensory activation primarily reflects the first stage of mismatch processing underlying the MMN, while parietal generators are involved at later stages (Bekinschtein et al., 2009; Phillips et al., 2016; Uhrig et al., 2014). Both prominent EEG mismatch signatures have been described as involuntary attention orienting responses to unexpected sensory input (Escera et al., 2000; Näätänen, 1992; Schröger et al., 2015; Wetzel & Schröger, 2014). The identified fronto‐parietal activations might therefore be reflective of the attention network identified by Corbetta and Shulman (2002) which has been suggested to orient attention to salient exogenous events and might represent a modality independent system for novelty alerting (Corbetta et al., 2008; Kim, 2014; Macaluso, 2010). The ventral part of the network includes IFJ, TPJ, IC and SMA/ACC (Corbetta & Shulman, 2002; Eckert et al., 2009; Yeo et al., 2011) and has been specifically associated with expectation violation (Vossel et al., 2014). As such, prior research highlights a central role for stimulus expectancy for an involvement of the identified network and suggests an interplay of expectation violation and attentional recruitment at later stages of mismatch processing.

Our PPI results indicate that increased projections from sensory regions to the modality general mismatch network contribute to the processing of regularity violation. Strikingly, we found that the left and the right seed regions similarly converged to clusters with right hemispheric dominance, in line with prior research on the auditory mismatch network (Dietz et al., 2014, 2021). As highlighted by a recent review, the larger TPJ area, including its dorsal extension around SMG into AG and IPS (Igelstrom & Graziano, 2017), is thought to code matches (left lateralized) and mismatches (right lateralized) between expected and actual events across sensory, motor and cognitive operations and keeps track of their statistical contingencies (Doricchi et al., 2022; Parr et al., 2023). With the current study, we provide further evidence for such wider TPJ activation as a common signature of mismatch effects across the senses in addition to the classically demonstrated involvement of the (right) pre‐frontal cortex in MMN generation. Previous MMR studies have indicated connectivity modulations within and between the identified regions in accordance with hierarchical predictive processing (Allen et al., 2016; Auksztulewicz & Friston, 2015; Chennu et al., 2016; El Karoui et al., 2015; Fardo et al., 2017; Garrido, Kilner, Stephan, & Friston, 2009; Phillips et al., 2015; Phillips et al., 2016; Uhrig et al., 2014). Our results complement these findings by showing the domain generality of connectivity modulations in the fronto‐parietal mismatch network underlying expectation violation. While PPI‐connectivity provides the advantage of an assumption‐free exploration of functional correlation within the brain with the seed regions, future studies would benefit from DCM analyses to provide further insights into the directed modulations between the nodes identified here across modalities.

### 4.3 Modulation of deviant responses by stimulus expectancy

Our parametric contrasts revealed that deviant responses within the identified mismatch network were increasing as a function of the length of the preceding standard stimulus train. The underlying sources of such deviance modulation during auditory, somatosensory and visual sequence processing have not been rendered by fMRI up to today, although indicated by similar EEG responses in different modalities (Baldeweg, 2006; Cowan et al., 1993; Gijsen et al., 2021; Grundei et al., 2023; Haenschel et al., 2005; Imada et al., 1993; Sams et al., 1983). While previous fMRI studies have shown that deviant responses increase with the relative mismatch magnitude in terms of deviant properties (Doeller et al., 2003; Opitz et al., 2002; Rinne et al., 2005), we provide additional evidence for a stimulus‐independent mismatch increase with prior stimulus repetitions which is comparable across the senses. Moreover, our results show that higher order sensory regions were most reflective of the parametric modulation, largely overlapping with the sensory clusters of the identified mismatch network. Although less pronounced, additional train length effects were found in the identified modality general regions such as IFJ, TPJ and SMA/ACC, which suggests that the fronto‐parietal recruitment during mismatch processing is not a binary process (e.g., attention switch or not), but rather related to the degree of expectancy induced by the prior stimulus train. Under predictive processing accounts of MMN generation (Friston, 2005; Garrido, Kilner, Stephan, & Friston, 2009; Stefanics et al., 2014), this modulation of the deviant response reflects an increasing prediction error to an established sensory regularity and increasing precision of the top‐down prediction to repeat the current stimulus train (Auksztulewicz et al., 2017; Auksztulewicz & Friston, 2016). Our finding of expectancy modulated mismatch responses in higher order sensory cortices aligns well with research in rats showing that prediction error responses to regularity violations increase along the auditory processing pathway (Parras et al., 2017) with error responses predominantly found in hierarchically higher auditory regions (Luo et al., 2023; Parras et al., 2021) and adaptation dominating in primary auditory cortex (Parras et al., 2021). The additional (reduced) modulation of frontal and parietal mismatch clusters might indicate projections of remaining prediction errors to the modality independent hubs of the mismatch network, as suggested by the results of our PPI analysis. Overall, our results provide evidence for highly comparable dynamics of deviant responses based on stimulus expectancy across modalities most pronounced in sensory specific regions of the mismatch network.

### 4.4 The parietal hub of the mismatch network reflects cross‐modal expectation violation

We used tri‐modal probabilistic sequences to create cross‐modal regularities by defining the transitions in one modality conditional on the configuration of the other two modalities. As such, stimulus transitions were more or less likely based on the multi‐modal context and thus rather predicted or mispredicted. With the cross‐modal predictability implicit in the sequences we go beyond any previous attempts to locate MMRs to multi‐modal sequences. Across sensory modalities, we identified a dorsal part of the larger TPJ area around IPS (Doricchi et al., 2022; Igelstrom & Graziano, 2017) to be particularly sensitive to cross‐modal expectation violation. This is striking given that the area is well known as a major connection hub for different senses (Damasio, 1989; Hagmann et al., 2008; Tomasi & Volkow, 2011), mapping multi‐modal inputs in both human and non‐human primates (Avillac et al., 2007; Sereno & Huang, 2014). This *convergence zone* for multi‐modal information integration (Damasio, 1989) has been proposed to provide a critical gateway to transform sensory information into cognitively relevant functions (Mesulam, 1998). Specifically, the extended TPJ area, including the IPS, forms the major parietal network hub for multi‐modal integration and higher‐order cognition (Igelstrom & Graziano, 2017) and is particularly known for coding unexpected events across a variety of sensory and cognitive processes (Doricchi et al., 2022). In line with these indications, our seed‐based PPI‐connectivity results show connectivity modulations from the sensory region of each modality to TPJ and IPS during mismatch processing. Therefore, our results suggest that the extended TPJ area forms an integrative processing hub in the mismatch network with IPS specifically signaling expectation violation based on cross‐modal contingencies.

In the hierarchical structure of the cortex, the parietal convergence zone is proposed to provide an amodal interface between bottom‐up sensory inputs and hierarchically higher levels (Seghier, 2013), such as the frontal cortex which is considered to transform accumulated context dependent sensory evidence from parietal cortex into choice (Erlich et al., 2015; Hanks et al., 2015). The IPS activation found here might indicate accumulation and integration of multi‐modal sensory evidence which is further projected to frontal cortex forming predictions about multi‐modal regularities and ultimately informing decision making. Support for such a role of the IPS in a hierarchy of multi‐modal perceptual inference comes from recent advances in research on Bayesian causal inference showing that reliability weighted sensory estimates are integrated in IPS and used in interaction with frontal areas to infer the hidden causes of sensory inputs (Cao et al., 2019; Noppeney, 2021; Rohe & Noppeney, 2015). Moreover, it has been suggested that the IPS is involved in a modality‐general representation of sequences (Planton & Dehaene, 2021) as it is found during regularity violations across different modalities and presentation formats (Planton & Dehaene, 2021; Wang et al., 2015; Wang et al., 2019) which is in line with our results.

In a previous study, using the same paradigm in EEG (Grundei et al., 2023), we reported an increase of the P3 to mispredicted trials, indicating sensitivity of the response to the predictive multi‐modal stimulus configuration. We showed that early and late MMRs in our EEG data were best explained by a Bayesian observer tracking stimulus transition probabilities and that more central and later responses around the P3 appeared to specifically track stimulus transitions across multiple modalities. In recent MMR research, the local–global paradigm has revealed that the early MMN and the later P3 reflect two hierarchical stages of mismatch processing signaling regularity violation on increasing levels of sequence complexity (Bekinschtein et al., 2009; Chennu et al., 2013; Chennu et al., 2016; Dehaene et al., 2015; Dürschmid et al., 2016; El Karoui et al., 2015; King et al., 2014; Niedernhuber et al., 2022; Shirazibeheshti et al., 2018; Wacongne et al., 2011): While the MMN is primarily sensitive to local regularities, such as basic stimulus repetition, the P3 is additionally sensitive to global regularities such as a repeating pattern over an extended period of time. In addition to global sequence monitoring with respect to temporal regularities, the P3 appears to additionally be sensitive to global sequence regularities in terms of the multi‐modal stimulus configuration (Grundei et al., 2023). Therefore, we suggest that the late P3 MMR might signal violation of global sequence contingencies on multiple spatio‐temporal scales. Given that the P3 has fronto‐parietal generators, including the TPJ around SMG and IPL (Linden, 2005; Polich, 2007), the fMRI results of the current study indicate a correspondence of the intraparietal cluster sensitive to cross‐modal expectation violation with our previous EEG results. Such an interpretation is supported by fMRI studies using the local–global paradigm which show parietal activations during the global MMR in humans (Bekinschtein et al., 2009) and in macaque monkey IPS (Uhrig et al., 2014). Taken together, the IPS appears to keep track of global cross‐modal sequence regularities, potentially by estimation of transition probabilities across modalities.

The current work provides evidence that the activation of fronto‐parietal network nodes to stimulus changes is rather independent of the input modality. In particular activation of the IPS shows sensitivity to multi‐modal probabilistic stimulus combinations, as we found it modulated by cross‐modal regularities. However, an alternative interpretation to cross‐modal learning cannot be ruled out, namely that the three synchronously presented stimuli are bound together to a tri‐modal object or triplet. If this was the case, the brain might extract the transition probabilities of tri‐modal states specified in the transition matrix (see Figure 1) rather than tracking each uni‐modal input stream and learning the cross‐modal regularities on top. While most previous MMN literature is focused on uni‐modal inputs, such different approaches of multi‐modal mismatch processing have by now not been broadly explored and our work motivates further research in this direction. Therefore, future modeling studies should directly compare observer models tracking different sequence properties to evaluate if and at which stage probabilistic uni‐modal inputs are integrated to, and represented as, combinations of multi‐modal objects.

The IPS is also integral part of the (dorsal) network of attentional control where it is found to be a marker of memory related expectation violation (O'Connor et al., 2010) provoking attention allocation related to uncertainty in information retrieval (Hutchinson & Turk‐Browne, 2012). In addition to the general activation of the attention network by unexpected events (Vossel et al., 2014), the IPS in particular might reflect expectations related to stored memory traces which further supports the idea that the area might operate on a global scale of sequence processing. Moreover, in the fronto‐parietal attention network the IPS is involved in the selection of sensory expectations in a multi‐dimensional environment with co‐existing contingencies (Ferrari et al., 2022; Leong et al., 2017; Niv et al., 2015), directly in line with our results.

Overall, our finding of an involvement of the dorsal TPJ around the IPS in cross‐modal expectation violation integrates well with current research converging to a modality‐general role for the extended TPJ region in signaling a divergence between expected and actual events at various scales (Doricchi et al., 2022). Moreover, we provide evidence for a novel parietal contribution to the multi‐modal mismatch network and suggest that the IPS tracks cross‐modal probabilistic associations during global sequence monitoring.

### 4.5 Conclusion

With the current study we substantiate previous evidence for a shared mismatch network across modalities, involving modality specific sensory cortices as well as modality independent inferior frontal and temporo‐parietal areas. Additionally, we demonstrated PPI‐connectivity modulations from sensory regions to common multi‐modal network hubs during mismatch processing and show that deviant responses within the network were modulated by local stimulus repetition, suggesting highly comparable organization of the computation of expectation violation across the senses. Moreover, hierarchically higher regions of the mismatch network in the extended TPJ area around IPS were identified to signal cross‐modal expectation violation and might keep track of global multi‐modal sequence regularities. Overall, these findings shed light on mismatch responses to multi‐modal probabilistic inputs in a shared cortical network of expectation violation.

## FUNDING INFORMATION

This work was supported by Berlin School of Mind and Brain, Humboldt Universität zu Berlin (Miro Grundei; http://www.mind-and-brain.de/home/). The funder had no role in study design, data collection and analysis, decision to publish, or preparation of the manuscript.

## CONFLICT OF INTEREST STATEMENT

The authors declare no conflicts of interest.

## Supporting information


**Data S1.** Supporting Information.

## Data Availability

The data that support the findings of this study are available on request from the corresponding author. The data are not publicly available due to privacy or ethical restrictions.
