# Supplementary material for: A multimodal cortical network of sensory expectation violation revealed by fMRI
Source: Hum Brain Mapp. 2023 Sep 18;44(17):5871–91. doi: 10.1002/hbm.26482 (PMC10619418; doi:10.1002/hbm.26482)
Supplement: Supplementary file 1 — Data S1. Supporting Information. [file HBM-44-5871-s001.pdf]

Supplementary Materials

Grundei, M., Schmidt, T.T. & Blankenburg, F. (2023). A multimodal cortical network of sensory expectation violation revealed by fMRI. *Human Brain Mapping*

Alternative cross-modal predictability GLM

An alternative version of the GLM to test for cross-modal predictability effects by means of contrasting congruent/incongruent trials was implemented as a control analysis to the predicted/mispredicted contrasts, including a control for trial number and train lengths. To this end, *congruent>incongruent* trials were contrasted on the subject level. Trials were labelled "congruent" if they followed a trial with "congruent other modalities", and v.v. for "incongruent" trials. The GLM consisted of separate regressors for standards and deviants of each modality and each condition. The regressors were balanced by randomly sampling the included trials such that the regressors contained the same number of congruent/incongruent trials and that these each included the same number of trials of each level of train lengths. In order to ensure randomized trial inclusion, 100 different GLMs were calculated for each participant with different random samples and the subject-level contrast images were averaged across samples. Subsequently, the average contrast images were analyzed at the group level as follows:

With regards to the deviants, in runs with sequence setting of condition C2 (congruent other modalities make a change less likely), stronger deviant responses were expected when a change followed a trial with congruent other modalities. Therefore, the subject level contrast (congruent>incongruent) was hypothesized to be positive in runs of C2. Equivalently, the same contrast was hypothesized to be negative for deviants in runs of C1. For deviants in runs of C3 (the unpredictable condition) no difference between deviants following congruent and incongruent trials was expected. With regards to the standards, in runs with sequence setting of condition C1, stronger responses were expected when a repetition followed a trial with congruent other modalities. Therefore, the subject level contrast (congruent>incongruent) was hypothesized to be positive in runs of C1. Equivalently, the same contrast was hypothesized to be negative for standards in runs of C2. For standards in runs of C3 no difference between deviants following congruent and incongruent trials was expected.

The results for the described contrasts are shown in Figure S1 and show significant effects for the hypothesized pattern in IPS for deviants and standards, whereas no significant differences were found for trials of C3. As such, the results overall confirm the indications discussed in the main manuscript for a role of IPS in cross-modal prediction.

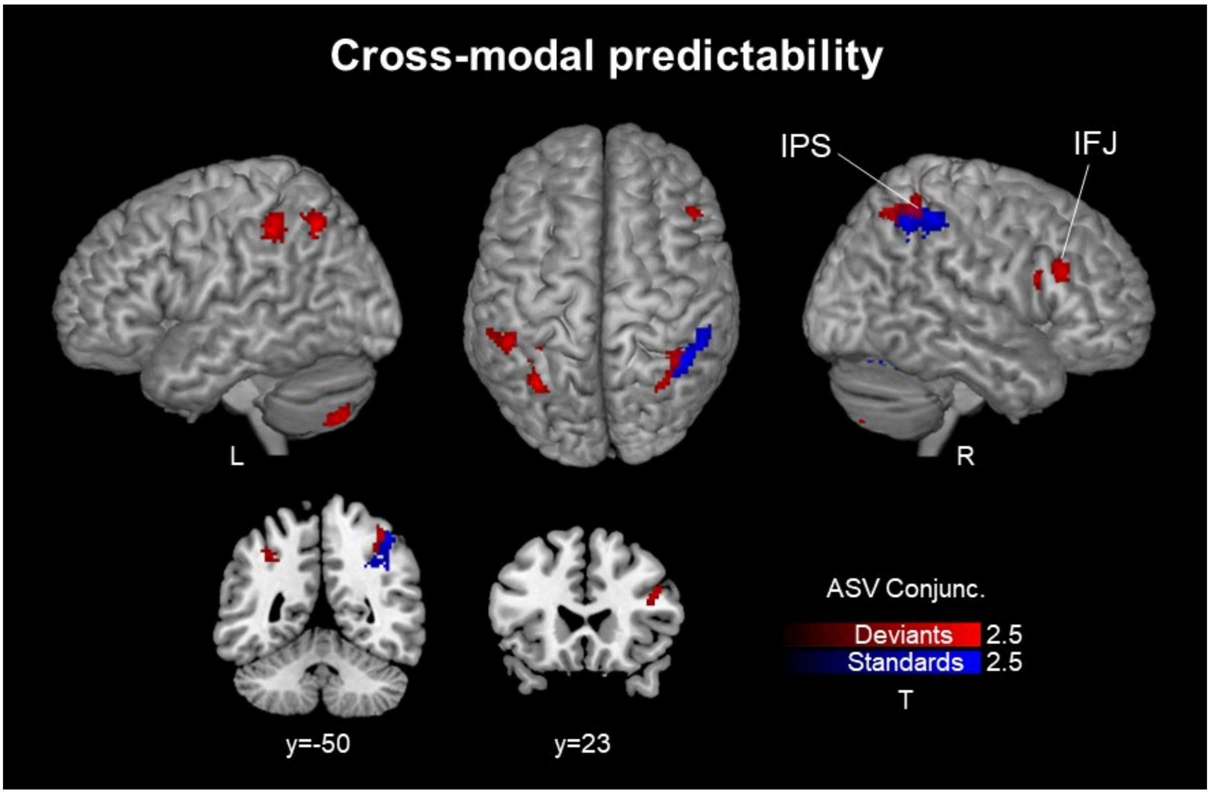

Figure S1: Results of alternative cross-modal predictability GLM. Significant clusters of activation across modalities (conjunction) for deviants (red) and standards (blue). Deviant contrast: congruent<incongruent in C1 and congruent>incongruent in C2. Standard contrasts: congruent>incongruent in C1 and congruent<incongruent in C2. $p<0.05$ FWE corrected on the cluster level. Unthresholded SPMs are available at https://www.neurovault.org/collections/LECDZXPI. Abbreviations of region labels: IFJ: inferior frontal junction; IPS: intraparietal sulcus.

Additional train length model with tri-modal regressor

In order to inspect repetition sensitivity of modality independent, tri-modal changes an additional train-length GLM was set up. This model consisted of a deviant regressor which was supplemented with parametric uni-modal train-lengths regressor for the deviants of each modality as well as an additional parametric regressor coding for tri-modal trains (with levels of 1, 2, 3, >3 prior repetitions), i.e., considering changes/repetitions *per se*, irrespective of the modality. This analysis firstly replicated the result of the train length model reported in the main manuscript (Figure 4) which correspond to uni-modal train length effects in the higher-order sensory regions for each modality. Further, the evaluation of the tri-modal train length regressor indicated the same regions as identified in the main analysis to show modality independent effects to sequence deviance (TPJ, preSMA, IFJ with right dominance, as shown in Figure 2 and similarly indicated for the conjunction of train length effects in Figure 4). These results provide some indication that the corresponding modality independent regions of the mismatch network might be sensitive to parametric modulation by sensory repetition *per se*.
